# Protein kinase C α enhances migration of breast cancer cells through FOXC2-mediated repression of p120-catenin

**DOI:** 10.1186/s12885-017-3827-y

**Published:** 2017-12-07

**Authors:** Thao N. D. Pham, Bethany E. Perez White, Huiping Zhao, Fariborz Mortazavi, Debra A. Tonetti

**Affiliations:** 1 Department of Biopharmaceutical Sciences, University of Illinois at Chicago, 833 South Wood Street M/C 865, Chicago, IL 60612 USA; 2 Division of Hematology/Oncology, Veterans Administration, Greater Los Angeles, University of California at Los Angeles, West Los Angeles, California USA; 3 Present Address:Division of Hematology/Oncology, Northwestern University, Chicago, IL USA; 4 Present Address: Department of Dermatology, Northwestern University, Chicago, IL USA

**Keywords:** Breast cancer metastasis, Protein kinase C, p120-catenin, FOXC2, Adherens junctions

## Abstract

**Background:**

Despite recent advances in the diagnosis and treatment of breast cancer, metastasis remains the main cause of death. Since migration of tumor cells is considered a prerequisite for tumor cell invasion and metastasis, a pressing goal in tumor biology has been to elucidate factors regulating their migratory activity. Protein kinase C alpha (PKCα) is a serine-threonine protein kinase implicated in cancer metastasis and associated with poor prognosis in breast cancer patients. In this study, we set out to define the signaling axis mediated by PKCα to promote breast cancer cell migration.

**Methods:**

Oncomine™ overexpression analysis was used to probe for *PRKCA* (PKCα) and *FOXC2* expression in mRNA datasets. The heat map of *PRKCA, FOXC2*, and *CTNND1* were obtained from the UC Santa Cruz platform. Survival data were obtained by PROGgene and available at http://www.compbio.iupui.edu/proggene. Markers for EMT and adherens junction were assessed by Western blotting and quantitative polymerase chain reaction. Effects of PKCα and FOXC2 on migration and invasion were assessed in vitro by transwell migration and invasion assays respectively. Cellular localization of E-cadherin and p120-catenin was determined by immunofluorescent staining. Promoter activity of p120-catenin was determined by dual luciferase assay using a previously validated p120-catenin reporter construct. Interaction between FOXC2 and p120-catenin promoter was verified by chromatin immunoprecipitation assay.

**Results:**

We determined that PKCα expression is necessary to maintain the migratory and invasive phenotype of both endocrine resistant and triple negative breast cancer cell lines. FOXC2 acts as a transcriptional repressor downstream of PKCα, and represses p120-catenin expression. Consequently, loss of p120-catenin leads to destabilization of E-cadherin at the adherens junction. Inhibition of either PKCα or FOXC2 is sufficient to rescue p120-catenin expression and trigger relocalization of p120-catenin and E-cadherin to the cell membrane, resulting in reduced tumor cell migration and invasion.

**Conclusions:**

Taken together, these results suggest that breast cancer metastasis may partially be controlled through PKCα/FOXC2-dependent repression of p120-catenin and highlight the potential for PKCα signal transduction networks to be targeted for the treatment of endocrine resistant and triple negative breast cancer.

**Electronic supplementary material:**

The online version of this article (10.1186/s12885-017-3827-y) contains supplementary material, which is available to authorized users.

## Background

Breast cancer is one of the most commonly diagnosed malignancies in women worldwide, according to the World Health Organization. Major advances in detection, diagnosis, and treatment have contributed to a steady decline in disease mortality [1]. However, metastasis remains the major cause of death in patients. At the molecular level, cancer metastasis is thought to be initiated by an epithelial-mesenchymal transition (EMT), a process whereby epithelial cells undergo drastic morphological and biochemical changes to acquire a spindle-shaped, highly motile, mesenchymal cell type [2, 3]. Loss of E-cadherin at the adherens junction (AJ) is considered a seminal and early event in EMT [2–4]. In cancer cells, down-regulation or loss of E-cadherin can result from inactivating mutations [5], promoter hypermethylation [6, 7], and transcriptional repression by EMT core regulators such as SNAIL [8, 9], ZEB [10], E12/47 [11], and TWIST [12]. p120-catenin, a cytoplasmic component of AJ, is a regulator of E-cadherin stability [13–15]. p120-catenin belongs to a family of armadillo-repeat proteins that binds to the highly conserved juxtamembrane domain of E-cadherin [16, 17]. Removal of p120-catenin or weakening E-cadherin-p120-catenin interactions can lead to rapid internalization and degradation of E-cadherin [13, 15, 18, 19]. Furthermore, loss of p120-catenin in lung cancer was shown to result in the transcription-independent reduction of E-cadherin [13, 20]. Therefore, factors that regulate p120-catenin can influence the stability of E-cadherin and AJs respectively. One of these factors is FOXC2, a forkhead transcription factor that actively represses p120-catenin transcription in non-small cell lung cancer (NSCLC) cell lines [20]. In this system, FOXC2-mediated repression of p120-catenin is causal to the down-regulation of E-cadherin protein [20]. In breast cancer, expression of FOXC2 is associated with and causal to chemotherapy resistance and metastasis in triple negative breast cancer (TNBC) [21, 22], a subtype defined by the absence of estrogen receptor (ER), progesterone receptor (PR), and human epidermal growth factor receptor 2 (HER2) expression. Yet, it remains unknown whether FOXC2 can actively repress transcription of p120-catenin in breast cancer.

Protein kinase C alpha (PKCα) belongs to the conventional subgroup of the PKC family that is comprised of 12 isozymes identified thus far [23–25]. Numerous studies, including our own, have demonstrated that expression of PKCα is associated with endocrine resistance [26, 27] and poor prognosis [27, 28] in ER-positive (ER^+^) breast tumors. In addition, expression of PKCα is elevated in TNBC patients [29, 30] and shown to be responsible for chemotherapy resistance and metastasis [30]. To the best of our knowledge, the relationship between PKCα and FOXC2 has not been examined.

In this study, we investigated the interplay among PKCα, FOXC2, and p120-catenin in breast cancer. We report a novel regulatory relationship between PKCα and FOXC2, particularly in endocrine resistant ER^+^ and basal A TNBC. Defined by microarray-based gene expression, basal A cell lines are distinct from basal B cell lines in that they are enriched in basal cytokeratins, ETS pathways and BRCA1 signatures [31, 32]. In basal A TNBC and endocrine resistant ER^+^ breast cancer, we demonstrate that PKCα is an upstream regulator of FOXC2 expression and activity. We report here that FOXC2 is a transcriptional repressor of p120-catenin leading to dissolution of AJs and enhanced migration and invasion in both ER^+^ and TNBC cell lines, events that potentially contribute to their metastatic potential.

## Methods

### Cell culture conditions and treatment

All cells were maintained in a humidified incubator with 5% CO_2_ at 37 °C. MCF7 cells were originally obtained from the Michigan Cancer Foundation (Detroit, MI) in 1992 and T47D cells were originally obtained from ATCC in 1996; both cell lines were stored at early passage. T47D:A18, a hormone-responsive clone, has been described previously [33]. T47D:A18 and MCF7 cells were cultured in RPMI with 10% FBS. MCF7:TAM1 [34], MCF7/PKCα [35, 36], MCF7:5C [37] and T47D:C42 [33] are hormone-independent and endocrine-resistant clones that were previously described. MCF7:TAM1 and MCF7/PKCα were cultured in RPMI with 10% FBS supplemented with 4-hydroxytamoxifen (4-OHT, 10^−7^ M) and G418 (100 μg/mL) respectively^1^. MCF7:5C and T47D:C42 were cultured in phenol red-free RPMI with 10% charcoal stripped FBS [33, 37]. Before experiments, estrogen-dependent cell lines were stripped in phenol red free media for 3 days. TNBC cell lines HCC1937 (CRL 2336™) and HCC1143 (CRL 2321™) were obtained from ATCC (Manassas, VA, USA). They were cultured and passaged in RPMI with 10% FBS according to the ATCC’s instruction. The TNBC cell line MDA-MB-231 (CL#10A) was cultured in MEM supplemented with 10% FBS. All cell culture reagents were obtained from Life Technologies (Carlsbad, CA, USA). Cell lines were tested negative for Mycoplasma contamination (MycoAlertTM Mycoplasm Detection Kit, Lonza Ltd., Walkersville, MD, USA), and were authenticated using Short Tandem Repeat (STR) method by the Research Resource Center core at the University of Illinois at Chicago (Chicago, IL, USA) in 2016. For TPA treatment, cells were treated with 100 nM for 2 h before mRNA was collected and analyzed.

### Western blot

Whole cell extracts of cultured cells were prepared in lysis buffer (Cell Signaling Technology, Danvers, MA, USA) supplemented with the protease inhibitor phenylmethane sulfonyl fluoride (PMSF). Protein concentration was determined by the bicinchoninic acid assay (BCA) (Thermo Fisher Scientific, Waltham, MA, US) and separated on SDS-PAGE gel. The following antibodies and dilution factors were used: PKCα (1:200, Santa Cruz Biotechnology, Santa Cruz, CA, USA), E-cadherin (1:1000, Cell Signaling Technology, Danvers, MA, USA), p120-catenin (1:200, Santa Cruz Biotechnology, Santa Cruz, CA, USA), FOXC2 (1:1000, Abcam, Cambridge, MA, USA). β-actin (1:1000, Sigma-Aldrich, St. Louis, MO, USA) was used as loading control. Blocking agents were either 5% non-fat dry milk or 5% bovine serum albumin (BSA) depending on the specific antibody. Mouse and rabbit horseradish peroxidase-conjugated secondary antibodies were purchased from GE Healthcare Life Sciences (Pittsburgh, PA, USA) and used at a 1:2000 dilution factor. Images of blots were acquired on a Bio-Rad ChemiDoc System following incubation with SuperSignal West Dura luminol solution (Thermo Fisher Scientific, Waltham, MA, USA). Protein bands were quantified using densitometry measured in Quantity One (Bio-Rad, Hercules, CA, USA). When necessary, membrane was stripped using Restore Western Blot Stripping Buffer (Thermo Fisher Scientific, Waltham, MA, USA).

### Migration and invasion assays

Corning^*®*^ transwell inserts (Corning Inc., Corning, NY, USA) were used for the migration and invasion assays following the manufacturer’s instruction. For invasion assay, inserts were coated with reconstituted Corning^*®*^ Matrigel^*®*^ Growth Factor Reduced (GFR) Basement Membrane (Corning Inc., Corning, NY, USA) and incubated for 2 h at 37 °C. Cells (1 × 10^5^) were plated in the upper chamber and FBS was used as the chemoattractant in the bottom chamber. For the experiments that involved MCF7/PKCα, fibroblast-conditioned media was used as the chemoattractant instead because we found FBS to be inhibitory to their migration and invasion (data not shown). After overnight incubation, inserts were fixed in ice cold 100% methanol and stained with a 0.2% crystal violet/ 2% ethanol solution. Following staining, inserts were rinsed with water and allowed to air dry before imaging. Total number of migrated and invasive cells/well was counted with 100X total magnification light microscopy. At least four areas per well were counted and averaged for analysis. Graph represents the fold change of number of migrating or invading cells relative to the control as explained in the legend.

### Quantitative reverse transcriptase-PCR (qRT-PCR)

mRNA was extracted by Trizol^®^ reagent (Thermo Fisher Scientific, Waltham, MA, USA) and purified following the manufacturer’s instruction. mRNA was reverse transcribed using the High Capacity cDNA Reverse Transcription kit (Applied Biosystems, Foster City, CA, USA). Detection of transcripts was done using a SYBR green reaction mixture in the StepOne Plus Real Time PCR Machine (Applied Biosystems, Foster City, CA, USA) using the standard amplification and detection protocol. Primer sequences are shown in Table 1.

**Table 1:** qPCR primers used in this study

Transcript	Forward primer (5′-3′)	Reverse primer (5′-3′)
*ACTB*	ATCGTCCACCGCAAATGCTTCTA	AGCCATGCCAATCTCATCTTGTT
*CDH1*	CCAGAAACGGAGGCCTGAT	CTGGGACTCCACCTACAGAAAGTT
*CTNND1*	ATGTTTGCGAGGAAGCCGC	CGAGTGGTCCCATCATCTG
*FOXC2*	GCCTAAGGACCTGGTGAAGC	TTGACGAAGCACTCGTTGAG

### Small-interfering (si) RNA-mediated knockdown

Cells were transfected with 50 nM (C_f_) siRNA targeting PKCα or FOXC2 following the manufacturer’s instruction. PKCα siRNA was purchased from Dharmacon (Lafayette, CO) (ON-TARGET plus SMARTpool) and Sigma Aldrich (predesigned, lab-validated siRNA). FOXC2 siRNA was purchased from Dharmacon (Lafayette, CO) (ON-TARGET plus SMARTpool) and IDT (San Jose, CA, USA) (Dicer-substrate, lab-validated siRNAs). Media was changed 24 h following transfection and every 3–4 days for the duration of the experiment. Efficiency of siRNA knockdown was confirmed with either qRT-PCR or Western blot. siRNA sequences are shown in Table 2. Specificity of PKCα siRNA is shown in Additional file 1: Figure S1.

**Table 2:** Small-interfering RNA used in this study

Gene	Sequences
PRKCA (ON-TARGET plus SMARTpool)	UAAGGAACCACAAGCAGUA UUAUAGGGAUCUGAAGUUA GAAGGGUUCUCGUAUGUCA UCACUGCUCUAUGGACUUA
FOXC2 (ON-TARGET plus SMARTpool)	CCUACGACUGCACGAAAUA CCAACGUGCGGGAGAUGUU GGAUUGAGAACUCGACCCU GCGCCUAAGGACCUGGUGA
FOXC2 (Dicer-substrates)	#1 5′ CGACUGCACGAAAUACUGACGUGTC 3′ 5′ GACACGUCAGUAUUUCGUGCAGUCGUA 3′ #2 5′ GGUGGUGAUCAAGAGCGAGGCGGCG 3′ 5′ CGCCGCCUCGCUCUUGAUCACCACCUU 3′ #3 5′ ACAUCAUGACCCUGCGAACGUCGCC 3′ 5′ GGCGACGUUCGCAGGGUCAUGAUGUUC 3’

### Luciferase reporter activity assay

The p120-catenin short luciferase reporter construct was kindly provided by Dr. Fariborz Mortazavi (Department of Medicine, David Geffen School of Medicine, University of California, Los Angeles, CA). To assess p120-catenin promoter activation, cells were co-transfected with p120-catenin reporter construct and β-galactosidase using Lipofectamine ^®^ 2000 (Thermo Fisher Scientific, Waltham, MA, USA). Luciferase activity was measured using the Dual Luciferase Reporter Assay (Applied Biosystems, Foster City, CA) and normalized against the activity of β-galactosidase following the manufacturer’s instructions.

### Confocal microscopy

Cells (2–4 × 10^5^) were seeded on coverslips in 6 well plates to reach 80% confluence in 2 days. Cells were fixed by incubating with 4% paraformaldehyde in PBS, pH 7.4 for 10 min at room temperature, and washed three times with ice-cold PBS. Permeabilization was achieved with 0.1% Triton-100X in PBS for 1 min. After three PBS washes, cells were incubated with blocking buffer (10% normal goat serum (Cell Signaling Technology, Danvers, MA, USA) in 1X PBS) for 1 h at room temperature, followed by overnight incubation with primary antibody in a humidified chamber at 4 °C. On the next day, coverslips were rinsed three times with wash buffer (0.1% BSA in 1X PBS), followed by 1 h incubation with secondary antibody for 1 h at room temperature. The following antibodies were used: mouse E-cadherin (Cell Signaling Technology, Danvers, MA, USA), rabbit p120-catenin, rabbit PKCα (Santa Cruz Biotechnology, Santa Cruz, CA, USA), anti-rabbit IgG (H + L), F(ab’)_2_ Fragment (Alexa Fluor^®^ 488 Conjugate) (Cell Signaling Technology, Danvers, MA USA), anti-mouse IgG (H + L), F(ab’)_2_ Fragment (Alexa Fluor^®^ 555 Conjugate) (Cell Signaling, Danvers, MA, USA). Following the manufacturer, all antibodies were used at 1:100 dilution factor: primary antibodies were diluted in blocking buffer (10% normal goat serum/PBS) and secondary antibodies were diluted in dilution buffer (1% normal goat serum/PBS). Coverslips were then incubated with Prolong® Gold Antifade Reagent with DAPI (Cell Signaling, Danvers, MA, USA) overnight. Images were obtained by the Zeiss Laser Scanning Microscope (LSM) 710 at the Core Imaging Facility at the University of Illinois at Chicago (Chicago, IL, USA). Intensity quantification was done using ImageJ.

### Chromatin Immunoprecipitation (ChIP)

Protocol was optimized from a protocol previously described by Carey et al. [38]. Specifically, 80-100μg of chromatin was incubated with either FOXC2 (ChIP grade, Abcam, Cambridge, MA, USA) or the negative control IgG (Cell Signaling Technology, Danvers, MA, USA) overnight at 4 °C. The antibody-DNA complex was captured by Protein G Agarose/ Salmon Sperm DNA bead (Millipore, Billerica, MA, USA). DNA was purified and analyzed by qRT-PCR using the previously reported primers that recognize p120-catenin promoter region ((20) as shown below. Primers that recognize the upstream and downstream region from the reported binding site (+127 to +309) of FOXC2 on p120-catenin were used as negative controls Table 3.

**Table 3:** ChIP primers used in this study

	Forward primer (5′-3′)	Reverse primer (5′-3′)
p120 ChIP (+127 to +309)	GATCCCGAAAGGAGGAAGAG	CGACTTGCTTATCCTCCTTTTCCC
p120 non-specific 1 (−34 to +126)	GTACTTTGGCGGGGGAGATT	AGCAGGGCTGAAACCGATAC
p120 non-specific 2 (+407 to +481)	GGCTGACATCACTTAGGAAAGC	CTCTTCCTCCTTTCGGGATC

### Oncomine™ data mining

Oncomine™ (Compendium Bioscience, Ann Arbor, MI, USA) overexpression analysis was used to probe for *PRKCA* (PKCα) and *FOXC2* expression in mRNA datasets. *P* values less than 0.05 were considered significant.

### The cancer genome atlas (TCGA) gene expression

For the generation of *PRKCA, FOXC2*, and *CTNND1* heat map, the TCGA data, analyzed using the AgilentG4502A_07_3 array platform, were obtained from the UC Santa Cruz platform (https://genome-cancer.ucsc.edu). All samples were intrinsically classified by PAM50 assay and the expression of ER, PR, and HER2. They were then stratified based on the relative transcripts expression of the selected gene (*PRKCA*, *FOXC2*, and *CTNND1*).

### Statistical analysis

All analyses were performed using GraphPad Prism 6.0 software. One-way and two-way ANOVA followed by default post-test or t-tests were used when appropriate. Statistics with *P* values less than 0.05 were considered significant.

## Results

### TNBC tumors display high expression of PKCα and FOXC2, and low expression of p120-catenin

**Fig. 1:**
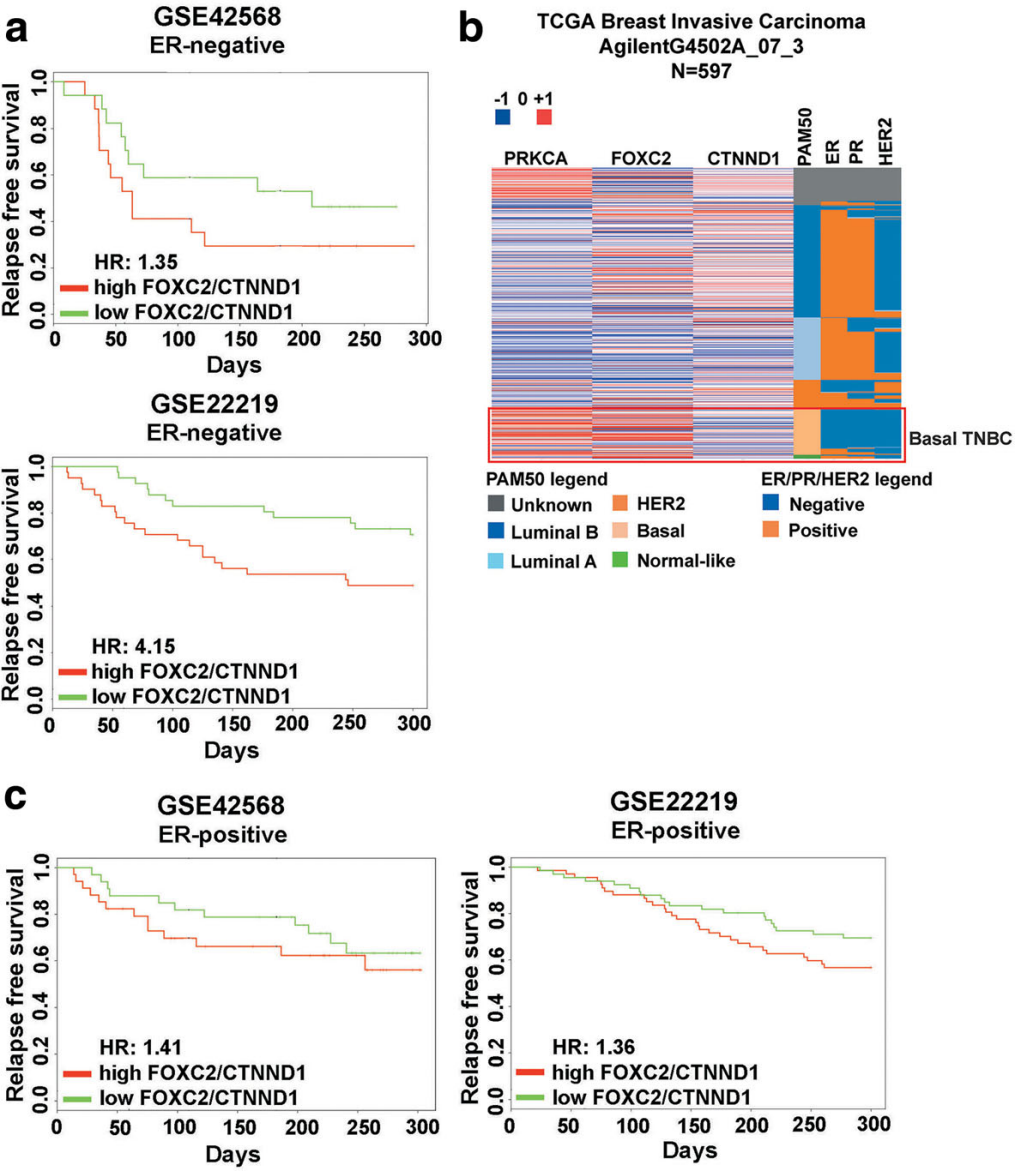
The PKCα - FOXC2 - p120-catenin pathway is prognostically relevant in breast cancer patients. **a** High expression of *FOXC2* and low expression of *CTNND1* (p120-catenin) in ER^−^ patients correlate with poorer relapse free survival (RFS). **b** TNBC/Basal-like patients express higher levels of *PRKCA* (PKCα), *FOXC2*, and lower levels of *CTNND1* compared to patients of other molecular subtypes. Molecular subtypes were determined by PAM50 assay. Gene expression data were computed and analyzed on UCSC Genome Browser (http://genome.ucsc.edu/). **c** High expression of *FOXC2* and low expression of *CTNND1* in ER^+^ patients are associated with a tendency towards poorer RFS. Survival data and significance were analyzed and obtained from PROGgene as previously described [55]

To investigate the relationship between PKCα, FOXC2 and p120-catenin, we first examined the Oncomine™ database for relative transcript levels of *PRKCA* (encoding for PKCα) and *FOXC2*. In four independent reports examining TNBC samples, both *PRKCA* and *FOXC2* rank among the top 10% of genes associated with the TNBC subtype (Additional file 2: Figure S2). These results are in agreement with previous reports that TNBC tumors express high PKCα [29, 30] and FOXC2 protein expression [21, 22]. Whereas FOXC2 was previously demonstrated to be a repressor of p120-catenin expression in lung cancer [20], it is not known whether this inverse relationship holds true in breast cancer. Kaplan-Meier analyses on two independent datasets, GSE22219 [39] and GSE42568 [40], support the hypothesis that in patients whose tumors lack ER expression, high *FOXC2*/*CTNND1* (p120-catenin) ratio (high *FOXC2*, low *CTNND1*) was associated with shorter relapse free survival (RFS) (*P* < 0.001 and *P* = 0.08 for GSE22219 and GSE42568 respectively) (Fig. 1a). Using microarray data provided by The Cancer Genome Atlas (TCGA) dataset, we were able to evaluate the expression levels of *PRKCA* (PKCα), *FOXC2*, and *CTNND1* in breast cancer patients (http://genome.ucsc.edu/). Patients were classified molecularly as normal-like, luminal B, luminal A, HER2-enriched, and basal-like [41], as well as by ER, PR, and HER2 expression. Relative expression of *PRKCA*, *FOXC2*, and *CTNND1* in all tumor samples, using the AgilentG4502A_07_3 array platform, were computed and visualized by a heat map. Overall, patients whose tumors are classified as basal-like and/or as TNBC express higher levels of *PRKCA* and *FOXC2* along with lower levels of *CTNND1* when compared to tumors of other subtypes (Fig. 1b). Interestingly, in the same GSE22219 and GSE42568 datasets, high *FOXC2*/*CTNND1* ratio also indicated a trend for reduced RFS for ER^+^ patients although the association is weaker than that in ER^−^ patients (*P* = 0.3 and 0.13 for GSE22219 and GSE42568 respectively) (Fig. 1c). Together, these data prompted us to further examine the functional consequence of the PKCα, FOXC2, and p120-catenin relationship in breast cancer at the molecular level.

### PKCα and its downstream target, FOXC2, enhance migration and invasion in basal A TNBC and endocrine resistant ER^+^ breast cancer

**Fig. 2:**
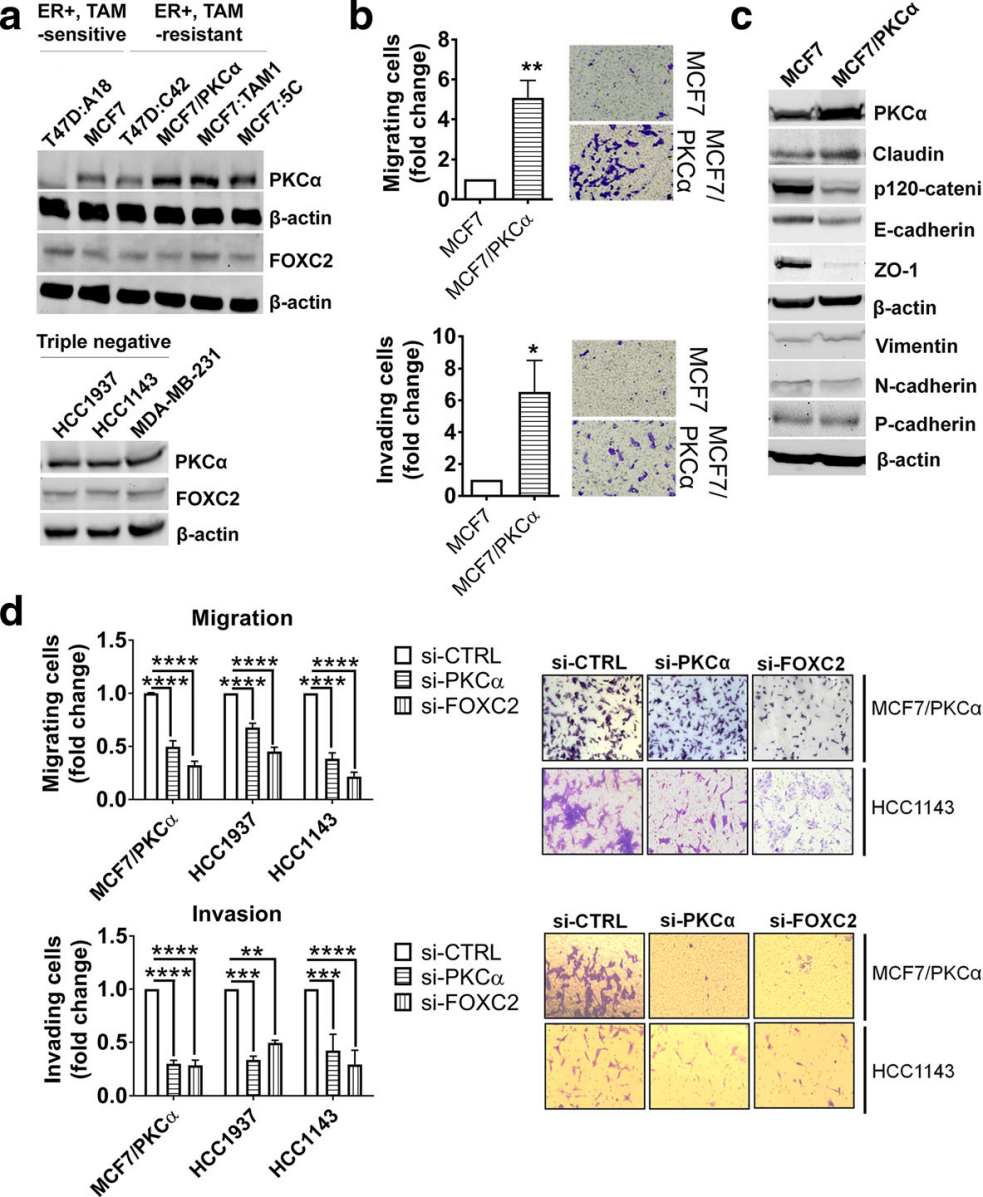
PKCα and FOXC2 enhance migratory and invasive capabilities of breast cancer cells. **a** Expression of PKCα and FOXC2 in a panel of breast cancer cell lines. **b** Migratory and invasive properties are assessed and compared between MCF7 and MCF7/PKCα. Representative pictures of migrating and invading cells are shown. **c** Expression of epithelial (ZO-1, E-cadherin, p120-catenin) and mesenchymal markers (Vimentin, N-cadherin, P-cadherin) in MCF7 and MCF7/PKCα are evaluated with Western blot. Blot is representative of three independent replicates. β-actin was used as the loading control. **d** Migration and invasion properties in breast cancer cells upon PKCα and FOXC2 knockdown. Experiments were done in the endocrine resistant cell line MCF7/PKCα and basal A TNBC cell lines HCC1143 and HCC1937. The number of migrating/invading cells per treatment was normalized against that of non-targeting siRNA treatment. Representative pictures of migrating and invading cells from MCF7/PKCα and HCC1143 cell lines are shown. Graphs represent the SEM of at least three independent biological replicates. Significance was determined by student t-test (**b**) and two-way ANOVA followed by Tukey’s test (**d**). *, *P* < 0.05 **, *P* < 0.01 ***, *P* < 0.001 ****, *P* < 0.0001

We examined the expression pattern of PKCα and FOXC2 in ER^+^ and TNBC breast cancer cell lines. Among ER^+^ cell lines, T47D:A18 and MCF7 cell lines are sensitive to endocrine treatment (such as tamoxifen) whereas T47D:C42, MCF7/PKCα, MCF7:TAM1 and MCF7:5C are all resistant to endocrine treatment as previously described [33, 34]. TNBC cell lines HCC1143 and HCC1937 (basal A) and MDA-MB-231 (basal B) were chosen based on molecular profiling [31, 32]. The basal B subgroup is reported to be highly enriched with EMT and stem cell signatures whereas basal A cell lines are characterized by upregulation of ETS- and BRCA-related pathways [31, 32]. Compared to basal B, the basal A subgroup is reported to better reflect the biology of the clinical basal-like breast cancer [31]. PKCα and FOXC2 are expressed in all endocrine resistant and basal TNBC (A and B) cell lines and among ER^+^ breast cancer cell lines, the endocrine resistant cells (T47D:C42, MCF7/PKCα, MCF7:TAM1, and MCF7:5C) have higher expression of PKCα compared to their endocrine sensitive counterparts T47D:A18 and MCF7 (Fig. 2a). When compared to their endocrine sensitive parental cell lines MCF7 and T47D:A18, MCF7/PKCα and T47D:C42 are more migratory and MCF7/PKCα cells are more invasive compared to MCF7 (Fig. 2b and Additional file 3: Figure S3). Interestingly, characteristics of enhanced migration and invasion observed in MCF7/PKCα partially correlate with markers consistent with an EMT (Fig. 2c). Specifically, MC7/PKCα cells show down-regulation of epithelial markers ZO-1 and E-cadherin compared to MCF7, however, elevated expression of mesenchymal markers Vimentin, N-cadherin, and P-cadherin is not observed (Fig. 2c). This result suggests that MCF7/PKCα cells have not undergone a complete EMT and perhaps this is not necessary for cancer cells to acquire a migratory and invasive phenotype.

Interestingly, either PKCα or FOXC2 knockdown was sufficient to reduce the migratory and invasive capabilities of MCF7/PKCα cells (Fig. 2d). Similarly, PKCα or FOXC2 knockdown in basal A cell lines HCC1937 and HCC1143 resulted in significantly lower migration and invasion capabilities (Fig. 2d). Therefore, we concluded that the positive contribution of PKCα and FOXC2 on migration and invasion can be extended beyond the scope of basal B TNBC [21, 22].

**Fig. 3:**
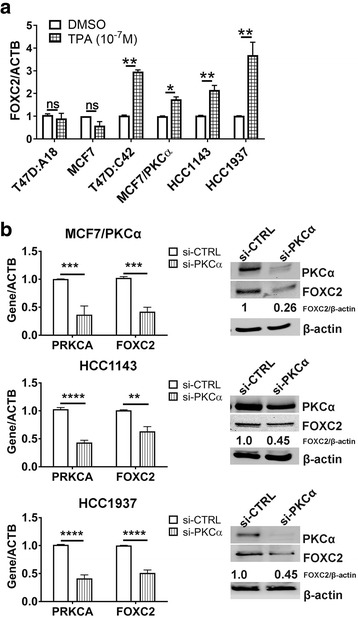
FOXC2 is a downstream target of PKCα. **a** Breast cancer cells were treated with either DMSO or TPA (100 nM, 2 h) and *FOXC2* expression levels were determined by qRT-PCR. **b** FOXC2 expression upon PKCα knockdown was assessed at both the transcript and protein level. Blots are representative of three independent replicates. β-actin was used as the loading control. Densitometry analysis of FOXC2 is shown. Graphs represent the SEM of at least three independent biological replicates. Significance was determined by student t-test and two-way ANOVA, followed by Tukey’s tests. *, *P* < 0.05 **, *P* < 0.01 ****, *P* < 0.0001

To assess a possible relationship between PKCα and FOXC2, all cell lines were treated with 12-*O*-tetradecanoylphorbol-13-acetate (TPA), an activator of several PKC family members, including PKCα. TPA treatment results in relocation of PKC isoforms from the cytoplasm to the cell membrane, indicative of activation. Indeed, upon TPA treatment, we observed a clear translocation of PKCα from the cytoplasm to the cell membrane in two representative cell lines (MCF7 and MCF7/PKCα) (Additional file 4: Figure S4). Following TPA treatment of endocrine resistant ER^+^ (T47D:C42, MCF7/PKCα) and basal A TNBC cell lines (HCC1143, HCC1937) we observed a significant induction of FOXC2 expression as measured by qRT-PCR (Fig. 3a). Correspondingly, PKCα knockdown using siRNA was sufficient to reduce FOXC2 expression at both the transcript and protein level in these cell lines (Fig. 3b). In contrast, TPA treatment did not have any effect on FOXC2 expression in either endocrine sensitive (T47D:A18, MCF7) (Fig. 3a) or basal B TNBC cell line (MDA-MB-231) (Additional file 5: Figure S5), suggesting a relationship between PKCα and FOXC2 in these two subtypes is unlikely. Altogether, our findings suggest that PKCα is a positive regulator of FOXC2 expression in endocrine resistant and basal A TNBC subgroups.

### Loss of PKCα can restore the AJ in endocrine-resistant breast cancer and TNBC cells

**Fig. 4:**
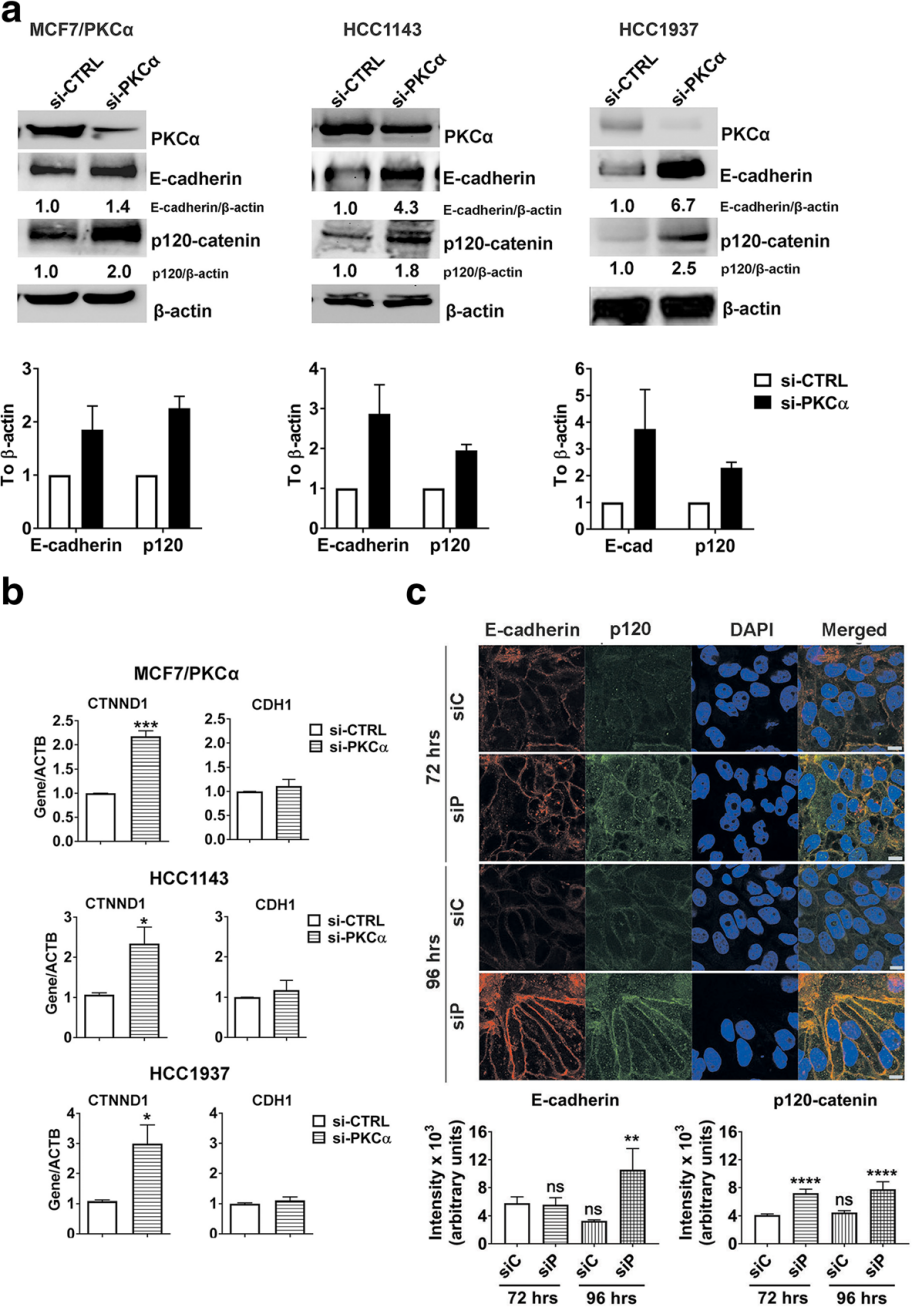
PKCα mediates transcriptional repression of p120-catenin and post-transcriptional repression of E-cadherin. **a** E-cadherin and p120-catenin protein expression upon PKCα knockdown was determined by Western blots. Blots are representative of three independent replicates. β-actin was used as the loading control. Densitometry analysis of E-cadherin and p120-catenin is shown. **b** E-cadherin (*CDH1*) and p120-catenin (*CTNND1*) expression upon PKCα knockdown was measured by qRT-PCR. Experiments were done in endocrine resistant breast cancer (MCF7/PKCα) and basal A TNBC cell lines (HCC1143, HCC1937). Graphs represent the SEM of at least three independent biological replicates. Significance was determined by student t-tests. **c** Membrane localization of p120-catenin and E-cadherin upon PKCα knockdown in HCC1143 was assessed by immunofluorescent staining according to Materials and Methods. Cells were treated with either negative siRNA (siC) or siRNA targeting PKCα (siP) and membrane localization of p120-catenin and E-cadherin was evaluated at 72 and 96 h after transfection. Scale bar 10uM. Quantification of p120-catenin and E-cadherin immunofluorescence intensity is shown. Significance was determined by one way ANOVA. *, *P* < 0.05, ** P < 0.01 ***, *P* < 0.001 ****, *P* < 0.0001

Loss of E-cadherin has been recognized as a characteristic of the transition from benign lesions to invasive, metastatic cancer [42]. At the molecular level, loss or reduction of E-cadherin expression precedes and is often causal to the dissociation of other members of the AJ, signifying the dissolution of intercellular adhesion [42, 43]. In agreement with the observation that PKCα enhances breast cancer cell motility (Fig. 2d), we examined the effect PKCα has on AJ components. PKCα knockdown resulted in a significant increase in E-cadherin and p120-catenin protein expression (Fig. 4a), suggesting that PKCα is a repressor of the two proteins. The increase of p120-catenin protein upon PKCα knockdown correlated with an increase in p120-catenin transcripts (Fig. 4b). However, no changes in E-cadherin transcripts were observed (Fig. 4b). This result suggests that E-cadherin repression by PKCα is not a transcriptional event and more likely a result from reduced protein stability. As loss of p120-catenin was previously reported to result in a transcription-independent reduction of E-cadherin [13, 20], we reasoned that PKCα-mediated repression of p120-catenin may be the underlying mechanism for E-cadherin loss. To address this hypothesis we examined p120-catenin and E-cadherin protein expression by immunofluorescent staining following PKCα knockdown. We determined that p120-catenin was recovered and localized at the cell membrane at 72 h after siRNA transfection, followed by a recovery of E-cadherin at approximately 24 h later (Fig. 4c). Quantitatively, we show that p120-catenin significantly recovered at an earlier time point than E-cadherin, supporting the notion that E-cadherin recovery is a downstream effect of p120-catenin recovery.

### FOXC2 is a transcriptional repressor of p120-catenin in endocrine resistant ER^+^ breast cancer and basal A TNBC

**Fig. 5:**
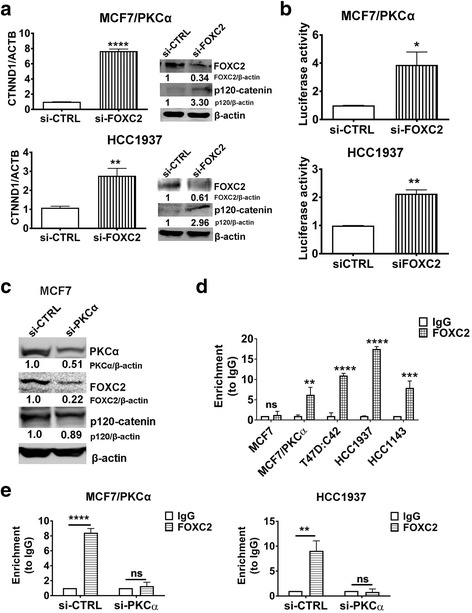
FOXC2 is a transcriptional repressor of p120-catenin. **a** Upon FOXC2 knockdown, p120-catenin expression at both the transcript and protein level was determined by qRT-PCR and Western blot respectively. Densitometry analysis for p120-catenin is shown. **b** The effect of FOXC2 knockdown on p120-catenin promoter activity was evaluated using a p120-catenin promoter luciferase reporter construct. **c** Expression of FOXC2 and p120-catenin protein upon PKCα knockdown in MCF7 cells was determined by Western blots. **d** FOXC2 binding to the p120-catenin promoter was determined by ChIP assay. **e** FOXC2 binding to the p120-catenin promoter with PKCα knockdown was determined by ChIP assay. Experiments were done in two representative cell lines in MCF7/PKCα (endocrine resistant) and HCC1937 (basal A TNBC). All blot images are representative of at least three independent biological replicates. β-actin was used as the loading control. Graphs represent the SEM of at least three independent biological replicates. Significance was determined by student t-test (**a**, **b**, **d**) and two-way ANOVA, followed by Tukey’s test (**e**). *, *P* < 0.05 **, *P* < 0.01 ****, *P* < 0.0001

FOXC2 was reported to be a transcriptional repressor of p120-catenin in NSCLC cell lines [20]. We sought to determine if the inverse relationship between FOXC2 and p120-catenin is also true in our breast cancer cell lines. FOXC2 knockdown in two representative cell lines, MCF7/PKCα and HCC1937, efficiently rescued p120-catenin expression at both the transcript and protein level (Fig. 5a). Furthermore, FOXC2 knockdown significantly increased the p120-catenin promoter activity as determined using a luciferase reporter construct (Fig. 5b). These data suggest that FOXC2 suppresses p120-catenin expression by repressing its transcription. It is noteworthy that PKCα knockdown did not result in recovery of p120-catenin expression in either MCF7 (Fig. 5c) or MDA-MB-231 (Additional file 5: Figure S5) even though both cell lines co-express PKCα and FOXC2 (Fig. 2a). Accordingly, we detected a significant enrichment of FOXC2 occupancy on the p120-catenin promoter in all cell lines representing either endocrine resistant or basal A breast cancer subtypes, but not in endocrine sensitive MCF7 (Fig. 5d). The interaction between FOXC2 and p120-catenin seems to take place within the +127 to +309 region of the p120-catenin promoter, as we were not able to detect FOXC2 binding either downstream or upstream from this region (Additional file 6: Figure S6). Finally, we found that FOXC2 binding to p120-catenin likely depends on PKCα expression because PKCα knockdown significantly reduced FOXC2 enrichment on p120-catenin (Fig. 5e). These findings cumulatively support the hypothesis that PKCα is a novel regulator of FOXC2-mediated repression of p120-catenin in breast cancer. Specifically, PKCα down-regulates p120-catenin by sustaining the expression and activity of FOXC2, a p120-catenin repressor. By repressing p120-catenin, PKCα promotes E-cadherin down-regulation and dissolution of the AJ, which impairs intercellular adhesion and promotes cellular migration. Specifically we found that this signaling axis is relevant in two breast cancer subtypes: endocrine resistant ER^+^ and basal A TNBC. Endocrine sensitive ER^+^ and basal B TNBC, despite being positive for PKCα and/or FOXC2, do not rely on PKCα for the repression of p120-catenin: PKCα knockdown in either MCF7 (endocrine sensitive) or MDA-MB-231 (basal B TNBC) was not sufficient to recover p120-catenin expression (Fig. 5c and Additional file 5: Figure S5).

## Discussion

**Fig. 6:**
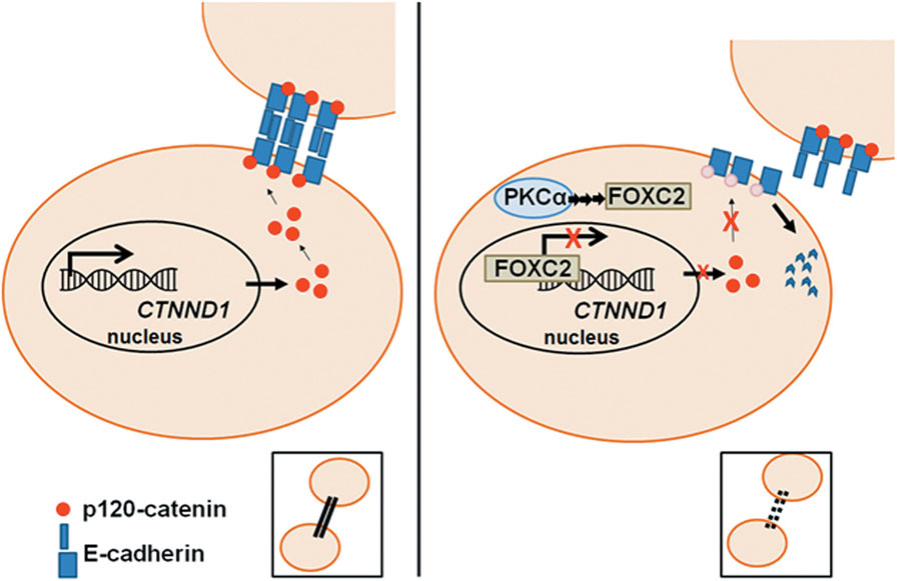
Signaling axis mediated by PKCα enhances cellular migration and invasion. In cells without PKCα expression (left), p120-catenin binds to the cytoplasmic domain of E-cadherin and stabilizes the AJs. In endocrine resistant ER^+^ breast cancer and basal A TNBC (right), PKCα increases FOXC2 expression and promotes its repression of p120-catenin transcription. As a result, E-cadherin is destabilized and prone to degradation, leading to dissociation of the AJ and intercellular connections

In the current study, we describe a novel signaling axis in endocrine resistant breast cancer and basal A TNBC involving PKCα, FOXC2, and p120-catenin that promotes cancer cell migration and invasion, which are considered integral steps in EMT. The schematic diagram summarizing the novel pathway is summarized in Fig. 6. E-cadherin is well-recognized as a tumor suppressor since loss of E-cadherin accelerates tumor formation and dissemination [44, 45]. Induction of E-cadherin in the aggressive, highly metastatic MDA-MB-231 breast cancer cells reduces their invasive ability in vitro [43] and in vivo [46]. The ability of p120-catenin to stabilize and maintain the expression of E-cadherin at the cell membrane suggests that p120-catenin itself may also be a tumor and metastasis suppressor [13–15]. In patients with invasive lobular carcinoma, partial or complete loss of membrane p120-catenin was associated with disease progression [47–50]. Down-regulation of p120-catenin is correlated with an increased risk of breast cancer-related death [49]. However, regulators of p120-catenin expression and modulators of its interaction with E-cadherin in breast cancer remain largely unknown. For the first time, we provide evidence to support the hypothesis that PKCα negatively impacts the AJ through FOXC2-mediated transcriptional repression of p120-catenin and subsequent destabilization and degradation of E-cadherin. Our reported findings strongly suggest that inhibition of either PKCα or FOXC2 could potentially reduce metastatic events in these two subtypes of breast cancer. As migration and invasion assays do not fully reflect the complexities of the in vivo microenvironment, future animal work is needed to evaluate the contribution of this pathway in tumor progression and metastasis.

One novel aspect of this pathway is that it occurs independently of E-cadherin transcriptional down-regulation. PKCα and/or FOXC2 can initiate EMT independently of previously described EMT core regulators such as SNAIL, SLUG, and ZEB. As previously reported, PKCα can collaborate with these factors to maintain mesenchymal features of post-EMT stem-like cells [30]. In this report, we demonstrate the role of PKCα in breast cancer cells that still retain epithelial morphology. This is particularly interesting as the concept of collective migration, a process whereby cells do not undergo EMT and therefore do not possess post-EMT features, has become increasingly described as a prominent invasion mechanism for low-grade tumors [51]. A recent report by Westcott and colleagues suggested cells participating in collective invasion are not necessarily more mesenchymal than non-invading cells [52]. In fact, leading tumor cells that pave the migration path for follower cells were shown to be indeed less epithelial, evidenced by lower expression of epithelial cytokeratins (*KRT8* and/or *KRT18*) but are not more mesenchymal, a conclusion based on the expression levels of basal cytokeratins (*KRT5* and *KRT14*) and EMT related genes (e.g. *SNAI1*) [52]. These findings and our own together do not negate the contribution of EMT in cancer metastasis but imply that subpopulations of cells in a tumor mass can utilize different mechanisms for directed migration and invasion.

The two TNBC cell lines chosen in our study, HCC1143 and HCC1937, belong to the basal A subgroup under TNBC [32]. Their gene expression profiles are enriched for ETS pathway genes, a pathway associated with tumor invasion and metastasis [53]. Compared to the basal B subgroup, which includes the commonly used cell lines MDA-MB-231 and BT-549, gene expression profiles of basal A are more similar to the clinical basal-like tumors [31], suggesting that they may represent a more relevant model to study this particular tumor type. In basal B cell lines, both PKCα and FOXC2 are required for the maintenance of breast cancer stem cells and their in vivo tumorigenicity [21, 30]. However, we found no evidence of the PKCα - FOXC2 - p120-catenin signaling pathway in MDA-MB-231 (Additional file 5: Figure S5). Similar observations were seen in MCF7, an endocrine sensitive ER^+^ that expresses both PKCα and FOXC2. These observations suggest that endocrine sensitive and basal B TNBC may rely on other signaling pathways to control for the expression and function of AJ. As a result, targeting PKCα - FOXC2 - p120-catenin signaling pathway may be more meaningful for endocrine resistant and basal A TNBC subtypes.

Our data indicate that PKCα can regulate FOXC2 at the mRNA level (Fig. 3b). The exact underlying mechanism for this phenomenon is currently unknown. Interestingly, while examining PKCα localization following TPA treatment, strong nuclear punctates were observed in both MCF7 and MCF7/PKCα (Additional file 4: Figure S4), indicating that a nuclear function of PKCα is possible. Notably, nuclear translocation and functions of PKCα have already been reported in thyroid cancer [54]. Future studies that examine these possibilities in breast cancer are of great interest.

## Conclusions

In summary, we show evidence that PKCα is a key regulator of migration and invasion in endocrine resistant ER^+^ breast cancer and basal A TNBC, but not in other subtypes such as endocrine sensitive ER^+^. In both subtypes, PKCα acts as an upstream regulator of FOXC2, which in turn represses the expression of p120-catenin, an important component of AJ that acts as the anchor for E-cadherin. Our data suggest an alternative pathway for E-cadherin loss that is not a result of the classical well-described transcriptional repression involving EMT transcription factors. Despite the fact that we did not observe a clear EMT in our cell lines, we showed that the loss of E-cadherin is associated with enhanced motility and invasion. This new mechanism can be further examined to determine whether post-translational loss of E-cadherin is sufficient to instigate an EMT in other systems. Our finding does not dispute the well-established EMT pathway; instead, it is highly possible that both mechanisms can work in parallel to promote cancer cell migration and metastasis. Future attempts that focus on disrupting the interactions between PKCα and downstream targets may have important therapeutic implications.

## Additional files

Additional file 1: Figure S1. Expression of various PKC isoforms in MCF7/PKCα cells upon PKCα siRNA transfection was examined by Western blot. Graph represents densitometry of three independent experiments with error bars representing SEM. (TIFF 716 kb)

Additional file 2: Figure S2. Four independent studies from Oncomine™ were used to assess expression levels of *PRKCA* (PKCα) and *FOXC2* transcripts in TNBC samples. (TIFF 2869 kb)

Additional file 3: Figure S3. (**a**) Migratory property was evaluated and compared between T47D:A18 and T47D:C42. (**b**) Expression of EMT markers in the two cell lines was examined by Western blot. (**c**) Basal p120-catenin promoter activity was evaluated in T47D:A18 and T47D:C42 using a p120-catenin promoter luciferase reporter construct. (TIFF 968 kb)

Additional file 4: Figure S4. MCF7 and MCF7/PKCα cells were treated with 100 nM TPA for 2 h and PKCα localization was assessed by confocal microscopy as described in Materials and Methods. Scale bar 10uM. (TIFF 5758 kb)

Additional file 5: Figure S5. (**a**) Expression of PKCα and FOXC2 in the three TNBC cell lines (basal A: HCC1937 and HCC1143; basal B: MDA-MB-231) was examined by Western blot. (**b**) MDA-MB-231 cells were treated with TPA (100 nM, 2 h) and expression levels of *FOXC2* mRNA were examined by qRT-PCR. (**c**) Following PKCα knockdown, expression of FOXC2 and p120-catenin in MDA-MB-231 was examined by Western blot. (TIFF 1695 kb)

Additional file 6: Figure S6. FOXC2 binding on the p120-catenin promoter at three different segments was evaluated by ChIP assay. qRT-PCR primer sequences are provided in Table 3. Data obtained from HCC1937 cell lines. (TIFF 544 kb)

## Abbreviations

AJ: Adherens junction
EMT: Epithelial-mesenchymal transition
ERα: Estrogen receptor
HER2: Human epidermal growth factor receptor 2
PKCα: Protein kinase C alpha
TCGA: The cancer genome atlas
TNBC: Triple negative breast cancer

## Footnotes

1 Prior to STR testing, previous publications incorrectly identified these cell lines as T47D instead of MCF7
