# Supplementary figures and images for: Protein kinase C α enhances migration of breast cancer cells through FOXC2-mediated repression of p120-catenin
Source: BMC Cancer. 2017 Dec 7;17:832. doi: 10.1186/s12885-017-3827-y (PMC5719564; doi:10.1186/s12885-017-3827-y)

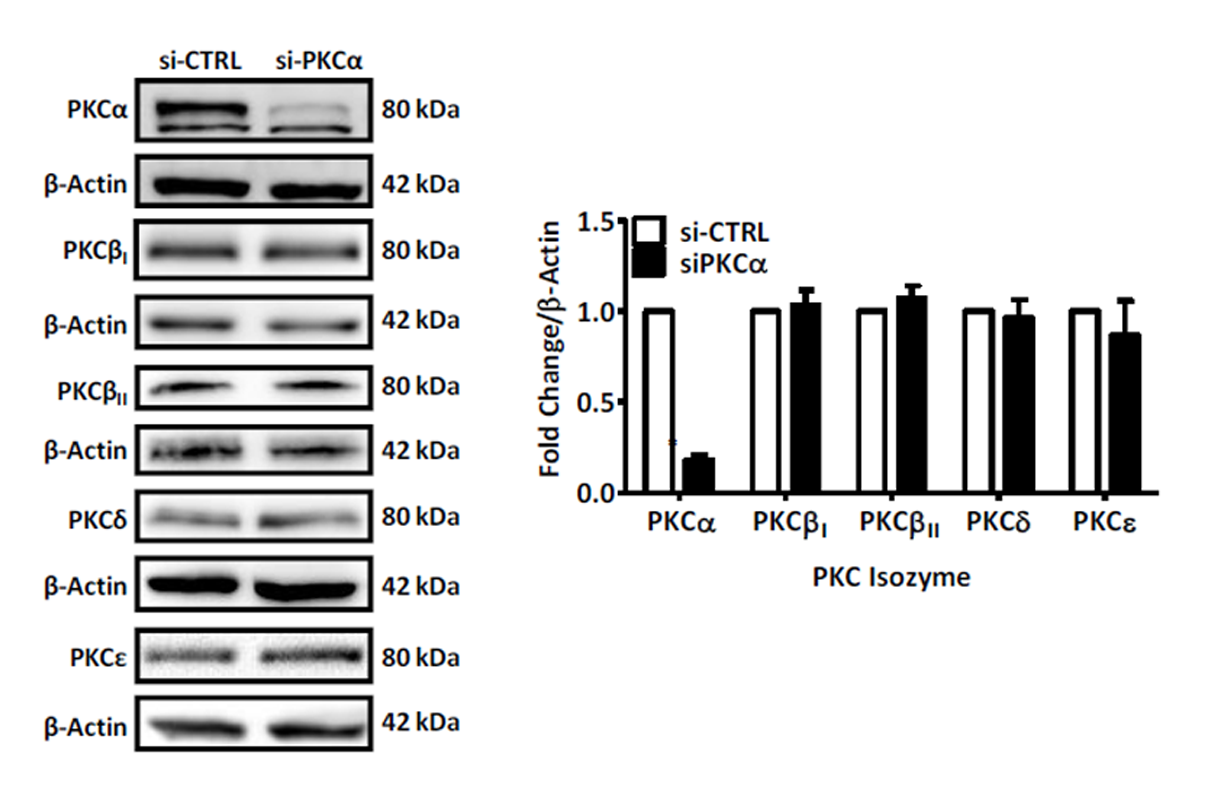

Supplement: Supplementary file 1 — Expression of various PKC isoforms in MCF7/PKCα cells upon PKCα siRNA transfection was examined by Western blot. Graph represents densitometry of three independent experiments with error bars representing SEM. (TIFF 716 kb) [file 12885_2017_3827_MOESM1_ESM.tif]

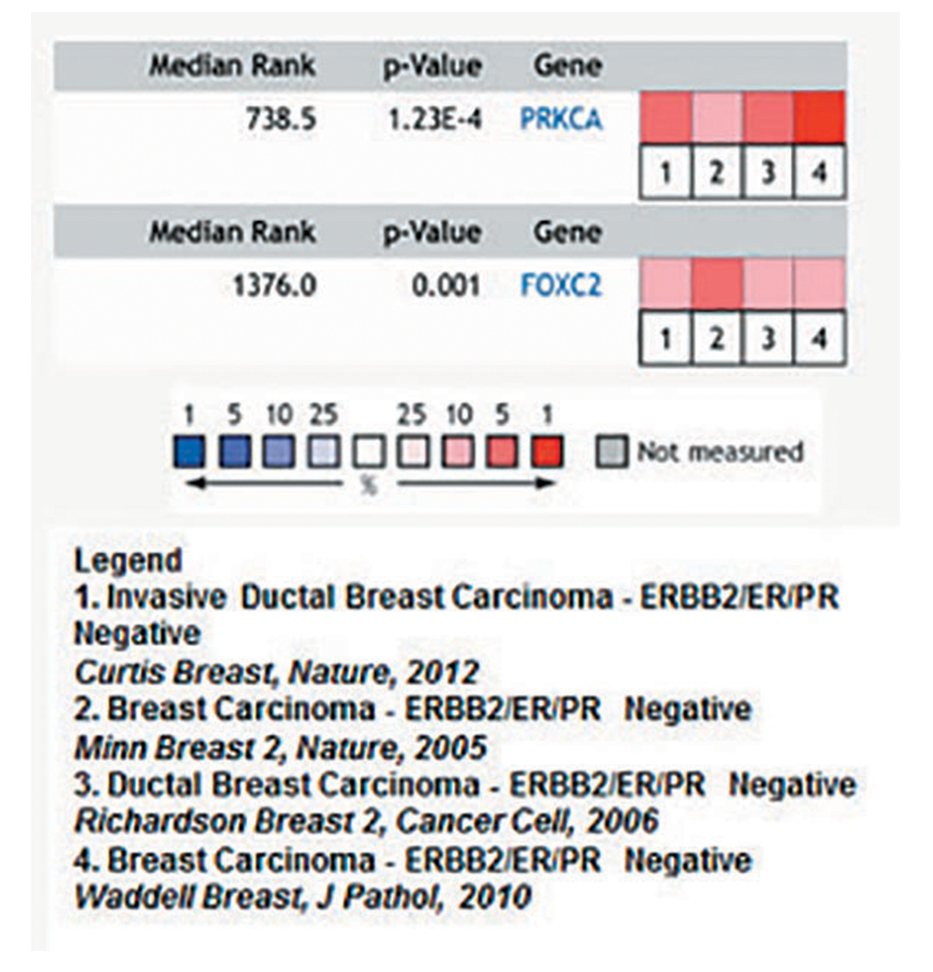

Supplement: Supplementary file 2 — Four independent studies from Oncomine™ were used to assess expression levels of PRKCA (PKCα) and FOXC2 transcripts in TNBC samples. (TIFF 2869 kb) [file 12885_2017_3827_MOESM2_ESM.tif]

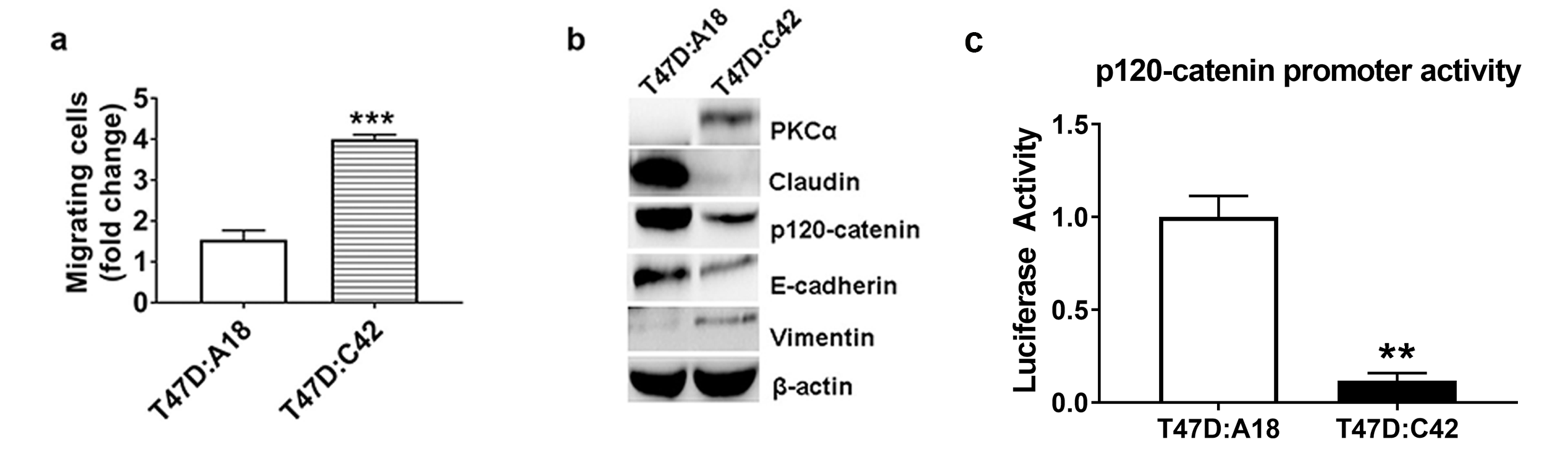

Supplement: Supplementary file 3 — (a) Migratory property was evaluated and compared between T47D:A18 and T47D:C42. (b) Expression of EMT markers in the two cell lines was examined by Western blot. (c) Basal p120-catenin promoter activity was evaluated in T47D:A18 and T47D:C42 using a p120-catenin promoter luciferase reporter construct. (TIFF 968 kb) [file 12885_2017_3827_MOESM3_ESM.tif]

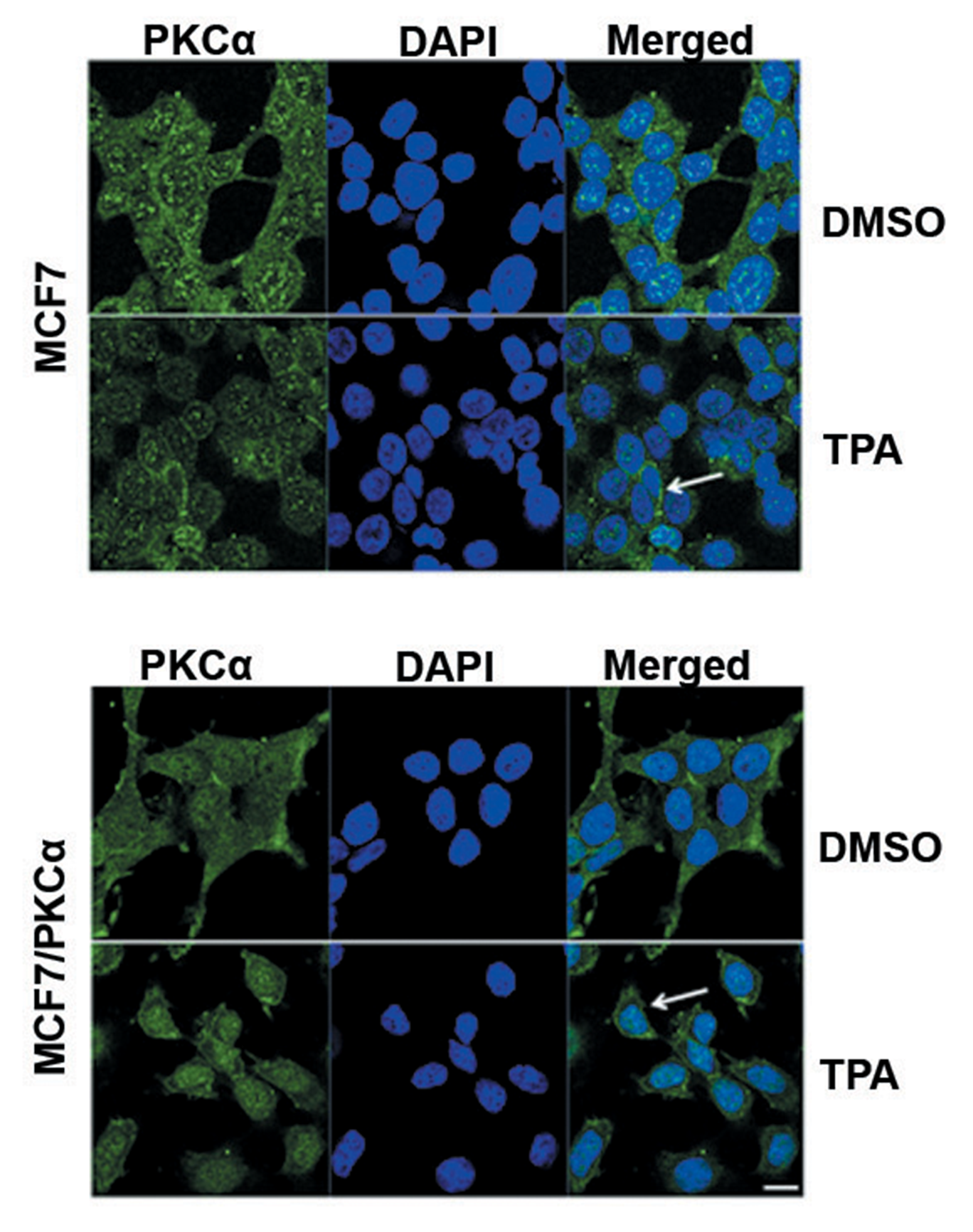

Supplement: Supplementary file 4 — MCF7 and MCF7/PKCα cells were treated with 100 nM TPA for 2 h and PKCα localization was assessed by confocal microscopy as described in Materials and Methods. Scale bar 10uM. (TIFF 5758 kb) [file 12885_2017_3827_MOESM4_ESM.tif]

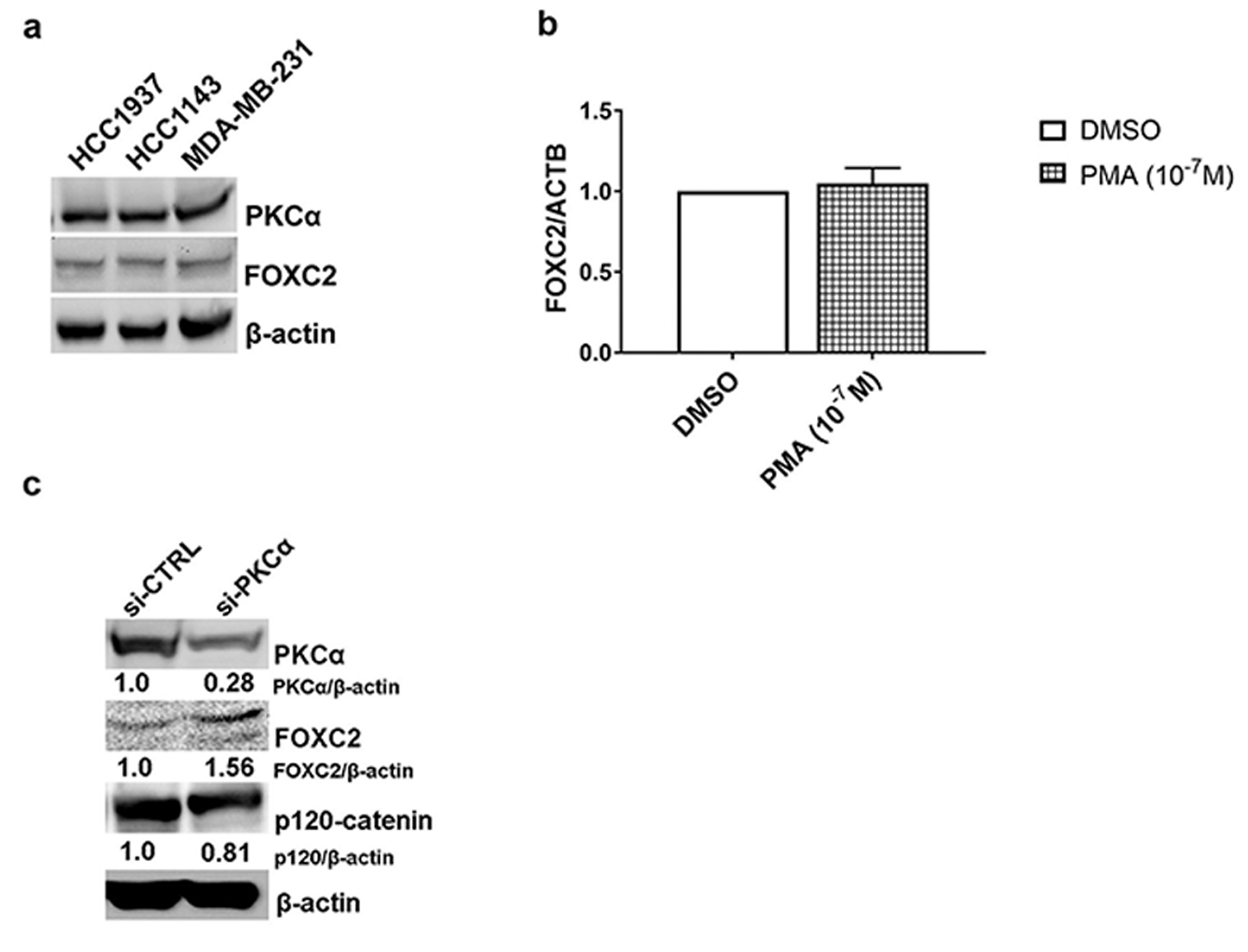

Supplement: Supplementary file 5 — (a) Expression of PKCα and FOXC2 in the three TNBC cell lines (basal A: HCC1937 and HCC1143; basal B: MDA-MB-231) was examined by Western blot. (b) MDA-MB-231 cells were treated with TPA (100 nM, 2 h) and expression levels of FOXC2 mRNA were examined by qRT-PCR. (c) Following PKCα knockdown, expression of FOXC2 and p120-catenin in MDA-MB-231 was examined by Western blot. (TIFF 1695 kb) [file 12885_2017_3827_MOESM5_ESM.tif]

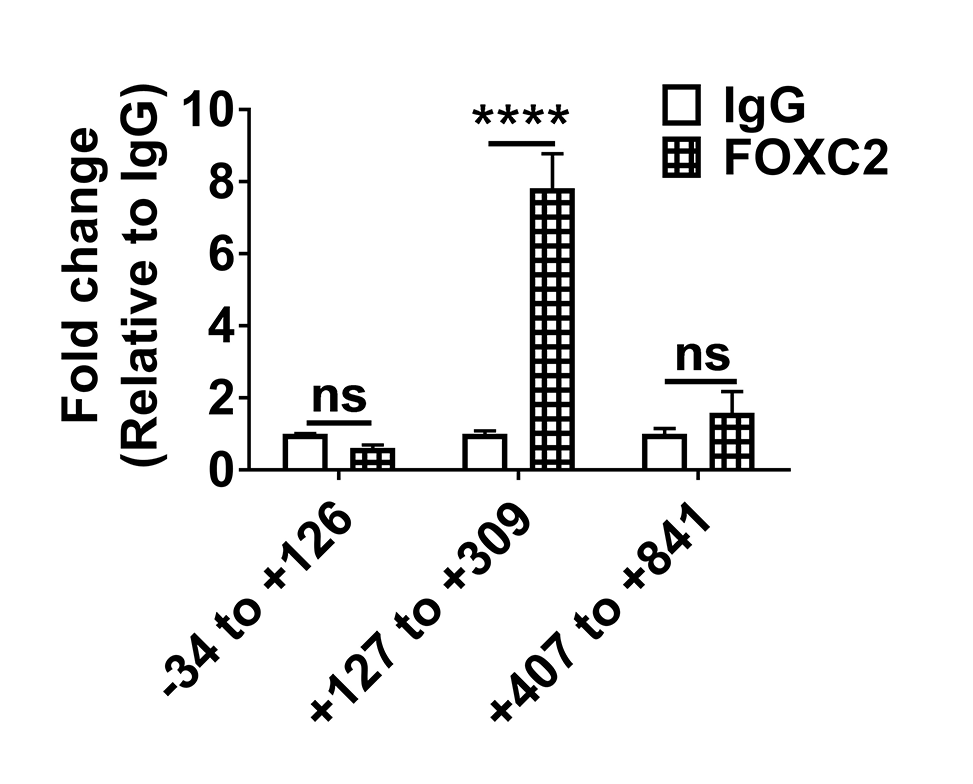

Supplement: Supplementary file 6 — FOXC2 binding on the p120-catenin promoter at three different segments was evaluated by ChIP assay. qRT-PCR primer sequences are provided in Table 3. Data obtained from HCC1937 cell lines. (TIFF 544 kb) [file 12885_2017_3827_MOESM6_ESM.tif]
